# Low dose post-transplant cyclophosphamide and sirolimus induce mixed chimerism with CTLA4-Ig or lymphocyte depletion in an MHC-mismatched murine allotransplantation model

**DOI:** 10.1038/s41409-024-02237-y

**Published:** 2024-02-12

**Authors:** Mariama D. Kabore, Corbin C. McElrath, Mohamed A. E. Ali, Katherine Almengo, Arunakumar Gangaplara, Cameron Fisher, Mauricio A. Barreto, Ahmad Shaikh, Purevdorj B. Olkhanud, Xin Xu, Deanna Gaskin, Maria Lopez-Ocasio, Ankit Saxena, J. Philip McCoy, Courtney D. Fitzhugh

**Affiliations:** 1 Cellular and Molecular Therapeutics Branch, National Heart, Lung, and Blood Institute, National Institutes of Health, Bethesda, MD 20892 USA; 2 Miltenyi Biotec, Gaithersburg, MD 20878 USA; 3 Department of Clinical Laboratory Sciences, College of Applied Medical Sciences, King Khalid University, Abha, Saudi Arabia; 4 Flow Cytometry Core, National Heart, Lung, and Blood Institute, National Institutes of Health, Bethesda, MD 20892 USA

**Keywords:** Bone marrow transplantation, Haematopoietic stem cells

## Abstract

Allogeneic hematopoietic cell transplantation (allo-HCT) offers a curative option for patients with certain non-malignant hematological diseases. High-dose post-transplant cyclophosphamide (PT-Cy) (200 mg/kg) and sirolimus (3 mg/kg), (HiC) synergistically induce stable mixed chimerism. Further, sirolimus and cytotoxic T lymphocyte-associated antigen-4 immunoglobulin (CTLA4-Ig), also known as Abatacept (Aba), promote immune tolerance and allograft survival. Here, in a major histocompatibility complex (MHC)-mismatched allo-HCT murine model, we combined Aba and/or T-cell depleting anti-Thy1.2 (Thy) with a lower dose of PT-Cy (50 mg/kg) and Sirolimus (3 mg/kg), (LoC). While mice in the LoC group showed graft rejection, the addition of Thy to LoC induced similar donor chimerism levels when compared to the HiC group. However, the addition of Aba to LoC led to graft acceptance only in younger mice. When Thy was added to the LoC+Aba setting, graft acceptance was restored in both age groups. Engrafted groups displayed significantly reduced frequencies of recipient-specific interferon-γ-producing T cells as well as an increased frequency in regulatory T cells (Tregs) except in the LoC+Aba group. Splenocytes from engrafted mice showed no proliferation upon restimulation with Balb/c stimulators. Collectively, in combination with Aba or Thy, LoC may be considered to reduce graft rejection in patients who undergo allo-HCT.

## Introduction

Allogeneic hematopoietic cell transplantation (Allo-HCT), which facilitates and maintains long-term immune tolerance, has tremendous potential to cure patients with non-malignant hematological diseases. While myeloablative conditioning regimens, which utilize high doses of total body irradiation (TBI) and/or chemotherapy to replace the recipient bone marrow with donor cells, may be highly effective, some patients may develop adverse clinical outcomes like graft-versus-host-disease (GVHD) [1]. Further, many adults experience excessive toxicities associated with myeloablative conditioning due to organ dysfunction caused by underlying chronic hematologic diseases or associated therapy [2].

Allo-HCT has been widely used to treat patients with malignant and nonmalignant hematological diseases, including hemoglobinopathies and primary immunodeficiency syndromes [3]. One of the significant differences in HCT between these two major categories is that mixed chimerism may be sufficient to reverse the non-malignant disease phenotype. For example, in patients with sickle cell disease (SCD), earlier studies involving the utilization of a myeloablative regimen illustrated that even persistent donor myeloid chimerism (DMC), seen in a minority of patients, resulted in a cure without SCD-related complications or GVHD [4–6]. Furthermore, we established, in a clinical setting, that only 20% DMC is needed to reverse the sickle phenotype based on vast differences in half-lives between donor (normal) and recipient (sickle) red blood cells (RBCs) [4].

Allo-HCT using a matched related or unrelated donor is a potential curative therapy for several non-malignant hematological disorders; however, the possibility of finding an HLA-matched donor is a major barrier to HCT [7]. Alternatively, HLA-haploidentical HCT (haplo-HCT) dramatically increases the donor pool relative to HLA-matched HCT [8]. The use of a major histocompatibility complex (MHC)-mismatched murine model of allo-HCT would mimic the mechanisms associated with host immune response, engraftment and graft failure in haplo-HCT settings [9].

Post transplant cyclophosphamide (PT-Cy) is an anti-neoplastic immunosuppressive drug widely used in allo-HCT. It decreases the incidence of GVHD [10] and causes alloreactive T-cell dysfunction [11]. PT-Cy in human HCT settings, used at high doses of 100 to 200 mg/kg, may be associated with adverse side effects, including nausea, cardiotoxicity, hemorrhagic cystitis, bacterial and fungal infection, and cytomegalovirus reactivation [12–14]. This highlights the cytotoxic nature of high-dose PT-Cy compared to its low-dose, as illustrated in prior studies [15–17]. Recently, others have shown an optimal cumulative dose of PT-Cy in mouse haplo-HCT to be 50 mg/kg [18–21]. PT-Cy at low doses is sufficient to cause alloreactive T cells to become functionally impaired in terms of their ability to cause GVHD and to proliferate [11]. Similarly, the addition of Sirolimus favors engraftment since it inhibits T cell activation through the blockade of mammalian target of rapamycin (mTOR) signaling and promoting Tregs [22, 23]. However, we previously reported graft rejection in mice given either high dose PT-Cy (200 mg/kg) or Sirolimus (3 mg/kg) in a mismatched murine model. However, a combination of high dose PT-Cy and Sirolimus for 15 days starting at day +4 PT resulted in successful engraftment, yet with slowly falling DMC levels from ~50% at one month to ~20% after 10 months post-HCT [18].

The addition of less cytotoxic immunosuppressive agents to conditioning regimens has enhanced the graft acceptance rate. CTLA4, expressed on CD4^+^ and CD8^+^ T cells, competes with CD28 to interact with co-stimulatory receptors (CD80 and CD86) on antigen-presenting cells (APCs). This interaction leads to checkpoint blockade through T cell unresponsiveness/anergy by the inability of CD28 to bind to CD80/CD86 [10]. Various transplant models have utilized this immune modulatory effect of CTLA4 to accommodate the graft through the chimeric version of this fusion protein, CTLA4-Ig (also known as Abatacept; Aba) which blocks T cell costimulation leading to T cell anergy, and when combined with Sirolimus, prolonged an orthotopic lung allograft in an MHC-mismatched rat model [24]. Lymphocyte-depleting agents are typically required with clinical haplo-HCT to prevent GVHD in patients [25]. In the murine transplant setting, anti-mouse Thy1.2 (Thy) can be utilized to deplete T lymphocytes to reduce graft rejection [26]. In addition, it has been reported that due to both intrinsic toxicities associated with PT-Cy and higher doses being associated with increased GVHD, the optimal dosage of PT-Cy would be 25 mg/kg/day at day three and day four post-HCT to prevent GVHD in murine allo-HCT [20]. We showed previously that high dose PT-Cy and sirolimus were synergistic; when employing both agents, donor myeloid chimerism (DMC) levels started at ~50% at one month PT and then slowly decreased to ~20% over time [18]. Here, we sought to determine if the addition of immunosuppressants Aba and/or Thy, combined with low dose of PT-Cy and Sirolimus (LoC) could increase donor chimerism levels and reduce regimen toxicity compared to high dose 200 mg/kg PT-Cy with Sirolimus (HiC).

Using a mismatched murine model, we evaluated the combinatorial effects of different immunosuppressants on transplant outcomes. Employing a mismatched murine model enabled us to evaluate different conditioning regimens that may facilitate long-term graft acceptance with minimal toxicities.

## Materials and methods

### Mice

Six-to-ten-week-old female Balb/c (H2-kd) donor, six-to-eighteen-week-old male and female C57Bl/6 J (H2-kb) recipient, and female C3H/HeJ (H2-kk) third-party mice were purchased from Jackson Laboratory (Bar Harbor, ME, USA). All mice were handled and cared for in accordance to the protocol, approved by the Animal Care and Use Committee at National Heart, Lung, and Blood Institute (NHLBI). All research conducted on animals followed biosafety level 2 guidelines. Following BMT, animals were monitored regularly for survival and morbidity (body weight, listlessness, ruffled fur). Mice who were unable to eat or drink and demonstrated persistent deterioration of body weight were euthanized.

### Bone marrow transplantation

Recipient C57Bl/6 J mice received 200 centigray (cGy) of total body irradiation (TBI) on the day of BMT. Femurs, tibias, pelvises, and the spine were dissected from euthanized donor Balb/C mice and cleaned of muscles and connective tissue on ice in 1x phosphate-buffered saline (PBS). Bone marrow cells were collected by crushing all the bones with a sterile mortar and pestle in FACS buffer (1x PBS containing 2% Fetal Bovine Serum (FBS)). RBCs were then lysed using either ammonium-chloride-potassium (ACK) lysing buffer (Quality Biological, Gaithersburg, MD, USA) or 10% sodium dextrose. BM cells were diluted in sterile 1x PBS. Each group of recipients received 20–25 × 10^6^ BM cells in 200 μl/mouse via retroorbital intravenous (i.v.) route. Following BMT, recipient mice were monitored for survival and signs of morbidity. Mice received neomycin-treated sterile water every week from day 0 to day 60 PT.

### Immunosuppressive therapy

Lyophilized Cy (Baxter, Deerfield, IL, USA), reconstituted with ultrapure water to make a stock solution of 20 mg/mL PT-Cy, was given intraperitoneally (i.p.) at a single dose of 200 mg/kg body weight/mouse on day 2 PT or one dose of 25 mg/kg body weight/mouse on day 3 and day 4 PT (cumulative total dose of 50 mg/kg). Sirolimus (Greenstone LLC, Peapack, NJ, USA) 1–2 mg tablets were crushed and suspended in carboxymethyl cellulose (Sigma-Aldrich, St Louis, MO, USA) and polysorbate 80 (Sigma-Aldrich) vehicle. Sirolimus was administered i.p., at a dose of 3 mg/kg body weight/day/mouse for 15 days as described previously (7). Anti-Thy1.2 monoclonal antibody (BioXcell, Lebanon, NH) was administered i.p. at 1 mg/mouse, i.p, in 200 μl of PBS on day −7 to day −4 pre-BMT. Lyophilized CTLA4-Ig (Bristol-Myers Squibb, Princeton, NJ, USA) was reconstituted in double distilled water and administered i.p., on days 2, 4, 6, 11, and 18 PT at 0.5 mg/100 μl per mouse.

### Mononuclear cell and lymphocyte isolation

Mononuclear cells (MNCs) and lymphocytes extracted from the peripheral blood and spleen, respectively, were prepared as described previously (10). Briefly, lympholyte (Cedarlane, Burlington, NC) was used to isolate MNCs from blood according to the manufacturer’s protocol. Spleens were harvested from mice on indicated days, and tissues were homogenized using a cell strainer (70 μm, Nest Scientific USA, Rahway, NJ). RBCs were lysed using a sterile ACK lysing buffer. Lymphocytes were washed and suspended in sterile RPMI complete medium supplemented with 10% heat-inactivated FBS, L-glutamine (2 mM), sodium pyruvate (1 mM), HEPES (1 mM), non-essential amino acids (0.1 mM), 2-mercaptoethanol (50 μM), and penicillin and streptomycin (100 U/ml), and the total amount of live cells was counted.

### Antibodies and flow cytometry

The antibody stain combinations were variable across trials and utilized based on lab user experience to provide minimal spillover between color detectors. All antibodies are specific to mouse species (BioLegend, San Diego, CA, BD Biosciences, San Jose, CA, or Thermo Fisher Scientific, Waltham MA). After isolation of spleen cells, peripheral blood MNCs, or both, cells (2-3 × 10^6^ cells) were suspended in a sterile complete medium. For surface staining, cells were stained in FACS staining buffer (PBS, 2% and heat-inactivated FBS). The antibodies used to detect donor-derived and recipient-derived murine antibody conjugates consisted of H2-kd and H2-kb, respectively. Myeloid cells were identified using anti-CD11b and anti-Gr1 antibodies, which delineate granulocytes, monocytes, macrophages, and dendritic cells (DCs). B cells were stained using an anti-CD19 antibody. anti-CD3 antibody used to stain mature T cells, CD4^+^Foxp3^-^ is used to identify helper T (Th) cells, Th2 and Th17 cells were identified via interleukin (IL-4)^+^, IL-17^+^ cytokines. anti-CD8 used to stain cytotoxic CD8 T cells. CD4^+^FoxP3^+^ population identified Tregs. DCs were identified using an anti-Cd11c antibody. NK cells were identified using an anti-CD49b and NK1.1 antibody. Macrophages were gated as the CD3^-^NK1.1^-^CD11b^+^ fraction. For intracellular staining cells were fixed, permeabilized and stained with interferon (IFN)-γ and TNF (tumor necrosis factor)-α antibodies. Cells were acquired in live^+^ population using LIVE/DEAD Fixable Aqua Dead Cell Stain Kit (Life Technologies, Carlsbad, CA). For intracellular detection of lag3 (C9B7W), GATA3 (L50-823), T-bet (4B10), Foxp3 (FJK-16s), and RORγt (B2D), cells were identified after fixation and permeabilization (Foxp3 Transcription Factor Buffer Set, eBioscience). Murine antibodies anti-Lag3, anti-GATA3 (BD Biosciences); anti-Foxp3, anti-RORγt, and anti-Tbet (BioLegend) were used. For intracellular cytokine detection, spleen cells (2-3 × 10^6^) in complete medium were stimulated with cell stimulation cocktail (ThermoFisher Scientific) containing PMA, ionomycin, Brefeldin A, and monensin for 5 h at 37^o^ C. The cells were then washed, fixed, permeabilized, and stained with intracellular cytokine antibodies anti-IFN-γ (XMG1.2), anti-IL-4 (11B11, BioLegend); anti-TNF-α (MP6-XT22), IL-10 (JES5-16E3), IL-17 (TC11-18H10, BD Biosciences); and TGF-β (R&D systems) overnight at 4^o^ C. Cells were washed and acquired by BD FACSymphony (BD Biosciences) flow cytometers with FASCDiva software. Flow cytometry data were analyzed using FlowJo Software (FlowJo LLC, Ashland, OR).

### Mixed lymphocyte reaction

Stimulator (donor group, Balb/C), third-party (C3H/HeJ), and responder (recipient group, C57Bl/6 J) spleen cells were obtained on week 28 PT as described above. Stimulator and third-party cells received 25 Gy irradiation and later, these cells were labeled with cell trace violet. Similarly, responder cells were labeled with CFSE according to the manufacturer’s protocol (ThermoFisher Scientific). Stimulator: responder or third-party: responder cells were plated at a 1:1 ratio, 2 × 10^5^ cells each in complete medium. Cells were incubated at 37 °C and on day 5 of culture, responder cells were analyzed for proliferation within CFSE^low^ CD4 and CD8 T cell populations by flow cytometry.

### Statistics

All statistical analyses of this study were carried out using GraphPad Prism software version 8–10 (GraphPad Software, Inc. La Jolla, CA). Differences in cell frequencies of groups were compared using an unpaired Student’s *t*-test. *P*-value < 0.05 was considered statistically significant. Data were presented as mean ± standard error of the mean (SEM).

## Results

### The addition of anti-Thy1.2 with or without Abatacept led to DMC levels of greater than 20% starting at week 10 PT

**Fig. 1:**
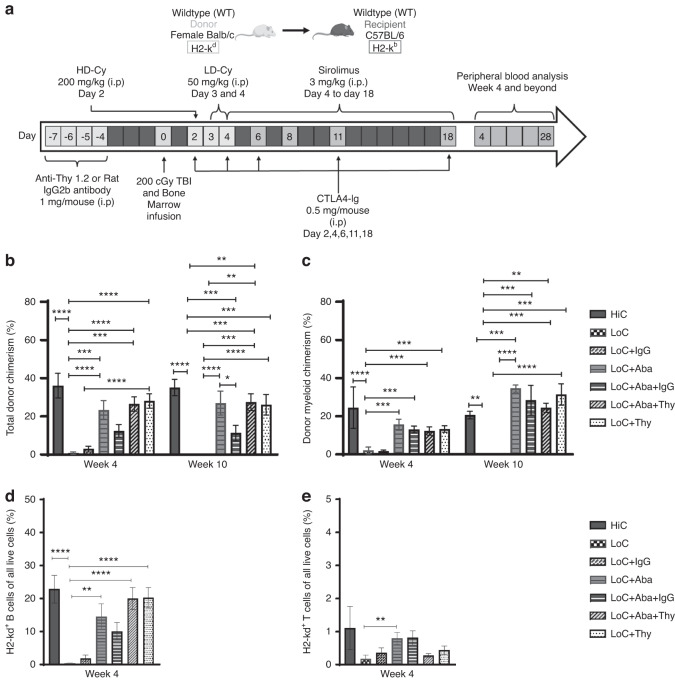
Thy1.2 with or without CTLA4-Ig led to total donor chimerism levels and donor myeloid chimerism levels of at least 20% from weeks 4–10 post-transplant. **a** The conditioning regimen details. 20 × 10^6^ Balb/c donor stem cells were transplanted into recipient WT B6 mice (*n* = 3–9 mice/group, 6–8 weeks old). Recipient mice received either PT-Cy (200 mg/kg single dose) on day +2 or PT-Cy (50 mg/kg cumulative dosing) on days +3 and +4. Mice were given anti-mouse Thy1.2 (1 mg/mouse, i.p) or Rat-IgG2b (1 mg/mouse, i.p.) from day −7 to day −4 before transplant. CTLA4-Ig was given (2 mg/mouse) on days +2, +4, +6, +11, and +18. Peripheral blood analysis at 4 and 10 weeks PT of total H2-kd^+^cells **b**, H2-kd^+^ CD11b^+^ myeloid cells **c**, H2-kd^+^ CD19^+^ B cells **d**, and H2-kd^+^ CD3^+^ T cells **e**. Data is obtained from *n* = 3–9 mice and represents two independent experiments (Mean ± SEM). D *P < 0.05, **P < 0.01, ***P < 0.001, and ****P < 0.0001 (unpaired two-tailed Student’s *t*-test).

We utilized a mismatched murine transplantation model in which female donor Balb/c BM cells were transplanted into male 7–8 week old C57Bl/6 J recipients. Recipient mice were conditioned according to the regimen detailed in Fig. 1a. Peripheral blood analysis showed that all mice conditioned with LoC rejected their graft (Fig. 1b, c) as early as week 4 PT, suggesting that insufficient immunosuppression led to graft rejection. Conversely mice that received Aba in addition to LoC (LoC+Aba) displayed improved total donor chimerism (TDC) and DMC levels (Fig. 1b, c) up to 28 weeks PT (Figs. S1a, b, S2a, b).

Next, we assessed the efficacy of combining Thy to LoC (LoC+Thy) with or without Aba. Thy given alone or in combination with Aba to mice which received LoC showed similar TDC levels compared to HiC (Fig. 1b, c) up to week 28 (Fig. S1a, b). These data show that the addition of Aba or Thy can provide sufficient immunosuppression to LoC to facilitate engraftment and maintain DMC levels close to or above 20% through 28 weeks PT.

We further evaluated innate and adaptive immune cells in peripheral blood based on donor (H2-kd) or recipient (H2-kb) MHC class I molecules to determine which cells contribute to engraftment. Donor cells in engrafted mice peripheral blood included H2-kd^+^ B cells from week 4, while T cell chimerism remained minimal (Fig. 1d, e). The same trend continues from weeks 10 through 28 (Fig. S1c, d). Our data are in tandem with prior studies demonstrating that myeloid and B cells contribute to most donor cells in engrafted mice [27, 28].

### Compared to rejected mice, a higher proportion of T regulatory cells to CD4^+^ and CD8^+^ T cells is observed in engrafted mice

**Fig. 2:**
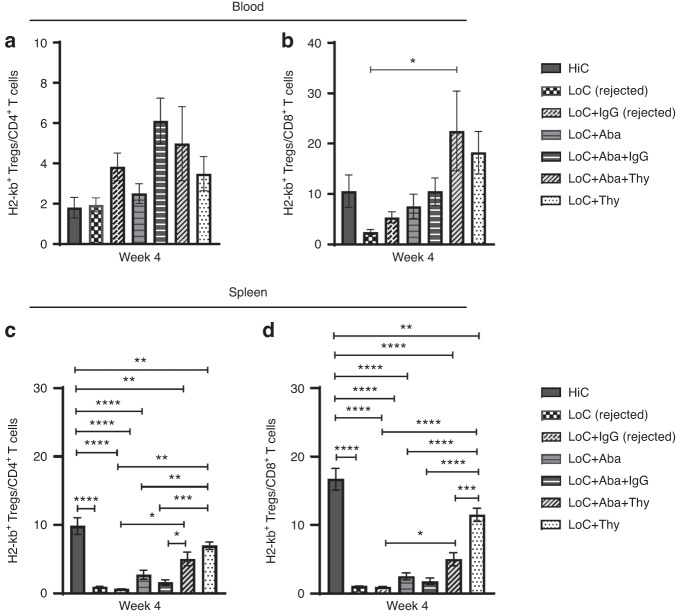
Recipient-derived Tregs compared to CD4 and CD8 T cells favor engraftment. Peripheral blood analysis at 4 weeks PT of host-derived H2-kb^+^ Tregs/CD4^+^ **a** and Tregs/CD8^+^ ratios **b**. Spleen analysis of host-derived H2-kb^+^ Tregs/CD4^+^ **c** and Tregs/CD8^+^ ratios **d**. Data represents two experiments from *n* = 3–9 mice (Mean ± SEM). *P < 0.05, **P < 0.01, ***P < 0.001, and ****P < 0.0001 (unpaired two-tailed Student’s *t*-test).

Although we did not find any clear correlation between the individual CD4^+^, CD8^+^ or Treg frequencies and the success or failure of engraftment in younger or older mice groups (Fig. S3a–c), peripheral blood analysis revealed that the recipient-derived Treg to CD4^+^ T cell ratio shows a modest trend with graft acceptance at week 4 PT (Fig. 2a, b). However, investigation of splenocytes revealed a significantly higher proportion of Treg to CD4^+^ or CD8^+^ T cells in successfully engrafted mice groups compared to rejected mice groups (Fig. 2c, d), particularly mice in HiC and LoC groups with additional Thy with or without Aba. Notably, the addition of Aba to LoC did not have a significant effect on Treg to CD4^+^ and CD8^+^ cell ratios (Fig. 2a–d). Representative flow plots of recipient derived CD4^+^ T cells, CD8^+^ T cells, and Tregs are shown in Fig. S3G.

### Recipient-derived regulatory T cells and Th2 cells detected in the spleen favor engraftment at week 4 PT

**Fig. 3:**
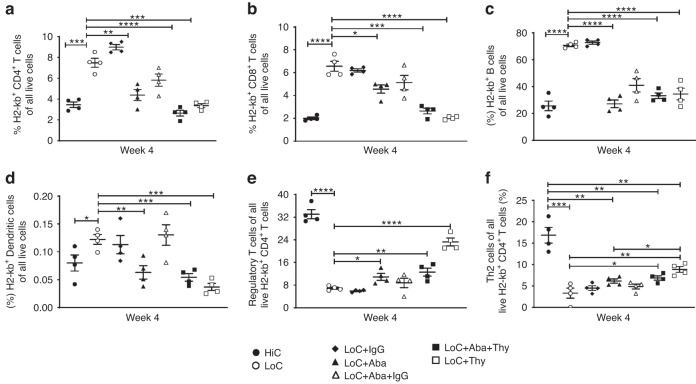
Analysis of early host-derived immune cell populations. Splenocyte analysis at 4 weeks PT of host-derived H2-kb^+^ CD4^+^ T cells **a**, H2-kb^+^ CD8^+^ T cells **b**, H2- kb^+^ CD19^+^ B cells **c**, H2-kb^+^ CD3^-^ CD19^-^ CD11c^+^ dendritic cells **d**, H2-kb^+^ CD4^+^ FoxP3^+^ Tregs **e**, and H2-kb^+^ CD4^+^ IL4^+^ Th2 cells **f**. Data shown from a representative experiment involving *n* = 4 mice (Mean ± SEM). *P < 0.05, **P < 0.01, ***P < 0.001, and ****P < 0.0001 (unpaired two-tailed Student’s *t*-test).

Others have reported that regulatory cells, including Tregs, type 1 regulatory (Tr1) cells, T helper (Th) 2 cells, and regulatory B cells (Bregs), mediate graft tolerance, survival, and function [29–32]. Paradoxically, inflammatory cells such as CD8^+^ T cells, CD4^+^ Th1 cells, Th17 cells, DCs, macrophages, and NK cells favor graft rejection [33, 34]. We observed only a negligible fraction of donor-derived T cells, therefore, we did not evaluate further. Next, we examined recipient-derived CD8^+^ and CD4^+^ T cells as well as other subsets of T cells, including Th1, Th2, Tregs, and Tr1 cells, NK cells, DCs, macrophages, B cells, and Bregs. We observed significantly higher frequencies of recipient-derived CD4^+^ (Fig. 3a), CD8^+^ (Fig. 3b), B cells (Fig. 3c) in rejected [LoC ± IgG] as compared to engrafted mice [HiC; LoC+Aba; LoC+Aba+Thy; LoC+Thy] splenocytes at week 4 PT. We also observed that donor-derived DCs were significantly lower in engrafted groups (Fig. 3d). In addition, when we analyzed recipient-derived CD4^+^ T cell subpopulations, we found that the CD4^+^Foxp3^+^ Tregs frequency was significantly elevated in engrafted mice (Fig. 3e). Likewise, CD4^+^ Gata3^+^ Th2 cell frequency was significantly elevated in most engrafted groups compared to rejected groups (Fig. 3f). These data suggest that recipient-derived Tregs and Th2 cells favor engraftment.

### Engrafted mice display reduced interferon- γ -producing CD4^+^ and CD8^+^ T cells and NK cells

**Fig. 4:**
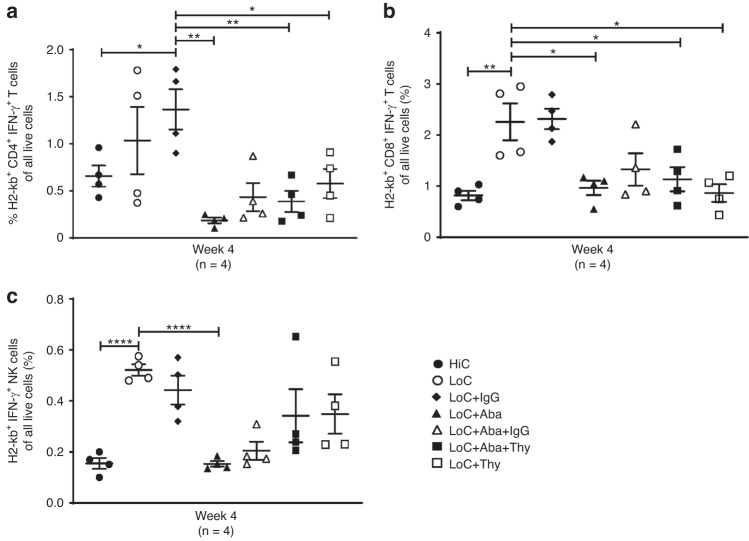
Engrafted mice display reduced IFN-g producing CD4^+^ and CD8^+^ T cells and NK cells. Splenocyte analysis at 4 weeks PT of host-derived H2- kb^+^ CD4^+^ IFN- γ^+^ T cells **a**, H2- kb^+^ CD8^+^ IFN- γ^+^ T cells **b**, and H2- kb^+^ CD4^+^ IFN- γ^+^ NK cells **c**. Data shown from a representative experiment involving *n* = 4 mice (Mean ± SEM). *P < 0.05, **P < 0.01, ***P < 0.001, and ****P < 0.0001 (unpaired two-tailed Student’s *t*-test).

Inflammatory cytokines such as interferon (IFN)-γ and tumor necrosis factor (TNF)-α produced by CD4^+^ and CD8^+^ T cells and NK cells play critical roles in allograft rejection in both mouse and human allotransplantation settings [35–38]. We, therefore, evaluated IFN-γ and TNF-α producing cells in our allo-HCT model and found that mice which rejected the graft [LoC ± IgG] illustrated an increased frequency in IFN-γ producing H2-kb^+^ CD4^+^ and CD8^+^ T cells compared with engrafted groups [HiC; LoC+Aba; LoC+Aba+Thy; LoC+Thy] (Fig. 4a, b). NK cells in engrafted Thy groups remained slightly elevated compared to remaining engrafted groups [HiC; LoC+Aba; LoC+Aba+IgG] (Fig. 4c). We did not find any substantial differences in TNF-α-producing cells between groups (data not shown). Next in our model, we evaluated the role of IL-10 and TGF-β; our data showed no statistical difference in IL-10 or TGF-β levels between engrafted and rejected mice (data not shown). These data show that IFN-γ-producing CD4^+^, CD8^+^ T cells, and NK cells are associated with graft rejection.

### Engrafted mice show functional donor spleen unresponsiveness upon restimulation

**Fig. 5:**
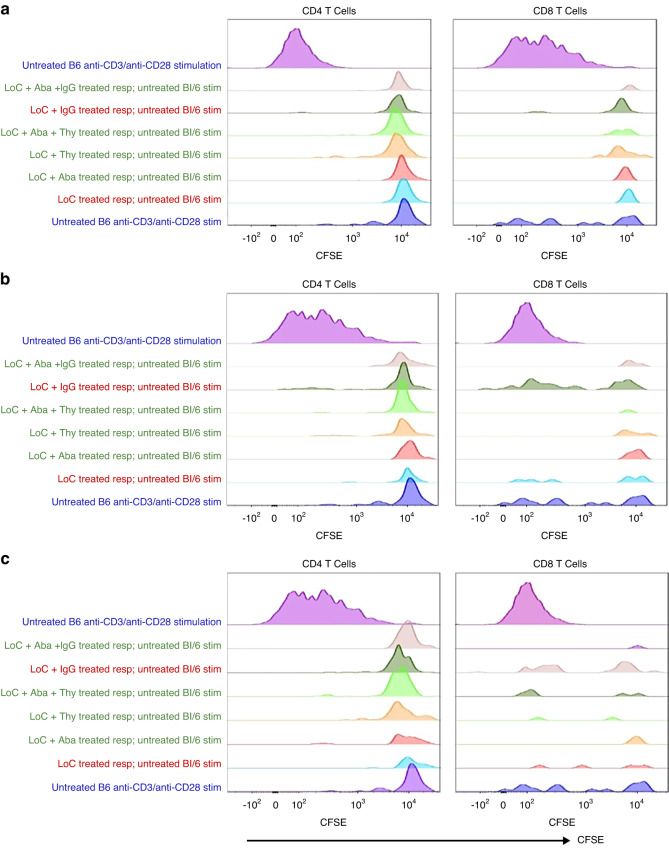
Functional immune tolerance assessment of host cells upon restimulation. MLR was performed on week-28 PT using responder splenocytes from LoC; LoC+Aba; LoC+Aba+Thy; LoC+Thy, LoC+IgG, LoC+Aba+IgG treated mice. Responder splenocytes were cultured with whole irradiated splenocytes obtained from C57Bl/6 mixed with self **a**, Balb/c **b**, and C3H/HeJ **c** (third-party**)** mice as described. CFSE^low^ proliferating CD4 and CD8 T cells were shown on day 5 post stimulation. Representative data from two experiments involving *n* = 5–10 mice are shown (Mean ± SEM).

Graft survival and function are commonly characterized by the persistence of donor unresponsiveness in allo-HCT settings. Hence, we examined the recipient mice splenocytes on week 28 PT in mixed lymphocyte reaction (MLR) culture conditions. Carboxyfluorescein succinimidyl ester (CFSE)-labeled responder splenocytes were mixed with either C57Bl/6 J (self), Balb/c (donor), or C3H/HeJ (third-party) irradiated stimulator cells. As expected, upon restimulation with recipient specific C57Bl/6 J splenocytes, neither engrafted nor rejected groups showed any proliferation in CD8+ and CD4^+^ T cells (Fig. 5a). Similarly, upon restimulation of CFSE-labeled responder splenocytes from engrafted mice with donor splenocytes, responder splenocytes showed no CD4^+^ and CD8^+^ T cell proliferation. On the other hand, rejected mice showed noticeable CD8 + T cell proliferation (CFSElow, Fig. 5b). As recipient mice had never been exposed to the third-party mice carrying the H2-kk^+^ haplotype, we observed that CD4^+^ and CD8^+^ T cells from all groups of recipient mice showed proliferation upon stimulation with third-party stimulators (Fig. 5c). These data demonstrate that recipient-derived T cells in engrafted mice are functionally unresponsive to donor antigen presenting cells (APCs).

### The addition of anti-Thy1.2 with or without Abatacept led to donor chimerism levels of approximately 30% to 40% in older mice groups

**Fig. 6:**
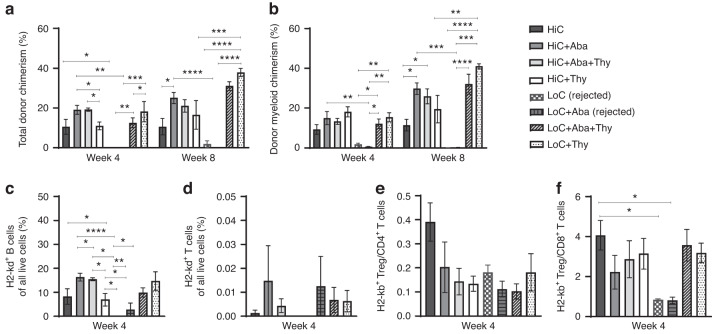
Thy1.2 with or without CTLA4-Ig led to total donor chimerism levels and donor myeloid chimerism levels of at least 20% from weeks 4 to 8 post-transplant in older mice. Peripheral blood at 4 and 8 weeks PT of total H2- kd^+^ cells **a**, H2- kd^+^ CD11b^+^ myeloid cells **b**, H2- kd^+^ CD19^+^ B cells **c**, H2- kd^+^ CD3^+^ T cells **d**, H2- kb^+^ Tregs/CD4^+^ **e** and Tregs/CD8^+^ ratios **f**. Representative data is depicted involving *n* = 5 mice (Mean ± SEM).**P < 0.01, ***P < 0.001, and ****P < 0.0001 (unpaired two-tailed Student’s *t*-test).

To delineate the potential effects of aging on transplant outcomes, we next analyzed an older group of male mice aged between 15 and 18 weeks at the time of transplant with the same conditioning regimen detailed in Fig. 1a. Similar to the younger male mice, older mice in HiC groups [HiC, HiC+Aba, HiC+Aba+Thy, HiC+Thy] showed successful engraftment. Interestingly, graft rejection was evident in older mice which received LoC with additional Aba [LoC+Aba] at weeks 4 and 8 PT (Fig. 6a and b) as well as in the spleen at week 12 PT (Fig. S4a, b). The addition of Thy with or without Aba to LoC, however, increased donor chimerism levels significantly in comparison to rejected groups. Representative flow plots of DMC and TDC are shown in Fig. S2c–d, respectively. Older male mice had a higher frequency of donor-derived B cells compared to T cells (Fig. 6c, d) that persisted in the spleen at week 12 PT (Fig. S4c, d). Lastly, at week 4 PT, although no differences in the individual CD4^+^, CD8^+^, Treg frequencies (Fig. S3d–f) or the Treg to CD4^+^ ratio (Fig. 6e) could be elucidated in the blood, there was a modest trend in engrafted mice achieving a higher ratio of Treg to CD8^+^ T cells in older male mice (Fig. 6f). Representative flow plots of recipient derived CD4^+^ T cells, CD8^+^ T cells, and Tregs are shown in Fig. S3H. These findings highlight the age-dependency of some immunosuppressant drugs that might fail to ensure successful engraftment in older recipients.

## Discussion

Our current results from young mice who received HiC showed DMC at ~20% at week 4 (Fig. 1c) and ~30% at week 28 (Fig. S1B), which is comparable to the results from our previous study in the long run [18]. Here, we report a novel conditioning regimen that allowed for similar levels of DMC using LoC, with the addition of Thy, with or without Aba, leading to sufficient engraftment levels. Further, donor chimerism levels were generally maintained above 20% through 28 weeks PT.

In both mouse age groups, we did not notice any synergistic effect on donor chimerism in mice given Aba and Thy in the LoC setting. However, while older mice administered Aba in the LoC setting rejected their grafts, the addition of Thy to mice given Aba in LoC groups did facilitate engraftment in both age groups. These results demonstrate that while Aba failed to achieve engraftment in older mice, the addition of lymphocyte depletion to LoC increased the efficacy of engraftment.

The sustained presence of Tregs is vital for graft acceptance due to their suppressive role on effector CD4^+^ T cell, cytotoxic CD8^+^ T cell, and APC function and cytokine production [39]. Expectedly, HiC yielded a higher proportion of recipient-derived Tregs to CD4^+^ or CD8^+^ T cells compared to LoC groups that rejected their grafts. The same trend was observed in mice engrafted in the LoC setting with an additional immunosuppressant of Thy with or without Aba [LoC+Thy, LoC+Aba+Thy]. The addition of Aba without Thy to LoC did not demonstrate significantly higher Treg to CD4^+^ and CD8^+^ T cell ratios, regardless of whether this drug combination was administered to younger mice that engrafted or the older mice that rejected their grafts. This suggests other mechanisms are at play for facilitating graft acceptance in mice administered Aba.

Previous studies have shown that the aging of recipient mice may alter the transplant course and impact underlying mechanisms related to graft acceptance and rejection [40–42]. As mice age, immunosenescence occurs, where changes in immune cell frequencies impact their function, which is further affected by immune suppression. One such example of age-related cellular change is the reduced effectiveness of P450, a hepatic cytochrome involved in the metabolism of drugs [40]. Due to the apparent age-dependent effects of older mice that may yield unforeseen mechanistic changes, we focused our mechanistic analyses on younger mice.

Cytokines such as interleukin (IL)-10, transforming growth factor (TGF)-β, and IL-35 producing Tregs and Th2 cells could facilitate immune suppression and can even determine graft outcome [43] Proinflammatory cytokines such as IFN-γ and TNF-α have been shown to play a pivotal role in graft rejection [44]. Sources of these cytokines include cells of the innate component, such as DCs, macrophages, and NK cells, and the adaptive component comprising CD4^+^ and CD8^+^ T cells [33, 45, 46]. Previous data illustrate that IFN-γ producing antigen-specific CD4^+^ and CD8^+^ T cells and non-antigen-specific NK cells mediate skin graft rejection as well as other models of both solid organ and HSC transplantation [35]. We have shown that recipient-derived IFN-γ producing CD4^+^ and CD8^+^ T cells and NK cells were increased in rejected groups. Earlier studies also noted the potential role of circulatory (plasma, serum, or both) IFN-γ in graft rejection [33]. These data strongly support that both cellular IFN-γ producing T and NK cells and soluble IFN-γ play predominant roles in graft failure. However, when we examined T cells producing proinflammatory (TNF-α, IL-17α) and anti-inflammatory (IL-4 and TGF-β) cytokines, which did not differ between the groups (data not shown), indicating that these cytokines may not have played a significant role in graft rejection with our conditioning regimens.

Previously, we have shown that the tolerance observed in engrafted mice who received a higher dose of PT-Cy and Sirolimus was also evident by recipient-derived unresponsiveness to donor APCs in vitro MLR [18]. When we performed the same analysis utilizing younger mice who received low-dose PT-Cy, Sirolimus, and Aba with or without Thy, donor hyporesponsiveness was unequivocally demonstrated in engrafted mice. Furthermore, engrafted groups that received Aba and Thy showed an immune response to third-party mice. These data suggest that in clinical settings, patients may be unresponsive to donor antigens while maintaining resistance to foreign pathogens.

Based on our findings, Aba plays a role in graft survival when combined with LoC in younger mice, yet minimal donor chimerism levels of 2.1% to clear graft rejection of 0% in older mice. Furthermore, Thy plays a role in facilitating higher levels of donor chimerism when combined with low dose cyclophosphamide and sirolimus. The observed graft rejection was due to IFN-γ-producing T cells and NK cells. Our data complement other studies which illustrate the role of IFN-γ signaling in mediating GVHD induction and the utility of monoclonal antibodies targeting IFN-γ, which may decrease the risk of graft rejection in allo-HCT models [47, 48].

Our study has limitations. Our selection of Thy in the conditioning regimen stemmed from our goal of mimicking lymphocyte-depleting drugs administered to patients on a clinical scale. For example, alemtuzumab and anti-thymocyte globulin (ATG) effectively deplete lymphocytes in patients with hemoglobinopathies [49, 50]. Additionally, prior studies have demonstrated CD90 to be a highly conserved domain in mice and humans [51]. However, the differential expression of CD90 and the impact of CD90 depletion on transplant outcomes has yet to be fully elucidated in mice compared to humans. As a result, a substantial limitation in our study is that Thy, alemtuzumab, and ATG act through different mechanisms. Nevertheless, our studies show the importance of employing lymphocyte depletion in conditioning regimens.

The difference in graft outcomes with mice administered the same conditioning regimen and with modest age differences remains unclear. Heinbokel et al., reported that Aba decreased cardiac and skin allograft survival in 18-month-old mice compared to 2-3-month-old mice. This difference was due to mechanistic evidence influenced by cellular changes in these two age groups. For instance, an increase in CD28-negative cells among CD4 + T cells in older mice led to inefficient targeting by Aba, manifesting as graft rejection [41]. This graft rejection was observed in tandem with increased IFN-gamma production in older mice administered Aba compared to younger mice [41]. Additionally, there was an increased expression of CD28 on Tregs in older mice recipients, which rendered old Tregs more susceptible to being targeted by Aba and, as a result, compromised the suppressor activity of Tregs in older mice [41]. However, in our study, the difference in recipient mice’s age during the time of transplant between younger (7–8 weeks) and older mice (15–18 weeks) was minimal. Regarding the effect of varying age on transplant outcomes, a significant question in our study remains about how a difference in age at the time of murine transplant led to varying engraftment outcomes. Although mice are considered young adult age at 3–6 months, middle age at 10–14 months, and older age at 18–24 months [52] based on Jackson Labs, there is no universally accepted standard for when mice are considered old. Indeed, a study demonstrated that adult mouse ages were inconsistent between studies and laboratories [53]. The age of mice considered adults ranged from 6 to 20 weeks [53]. These age differences can encompass ongoing developmental changes in a range of systems, which could profoundly impact the outcome of an experiment [53]. Another phenomenon that occurs in both mice and humans is immunosenescence – changes in the adaptive immune system over time. For example, a study notes a difference in naïve CD8 T cell frequencies in the blood of 7-8-week-old mice compared to 16-week-old mice [54]. Our study showed an age-dependent effect on engraftment outcomes in mice 6–8 weeks compared to 16–20 weeks. As a result, a limitation of our study was that these experiments were not done in middle or even old-age mice, as classified by Jackson Labs. Had this been the case, we could have elucidated clear mechanistic evidence for these differences in transplant outcomes. Nevertheless, our older mice showed a significant difference in the transplantation outcome with Aba conditioning, providing us with exciting avenues for future research.

We have reported a novel conditioning regimen that promotes donor chimerism without high dose cyclophosphamide. Together these data suggest that a mismatched murine model given low dose PT-Cy, sirolimus, and Thy, with or without Aba, can maintain DMC at levels close to or exceeding 20%. A clinical trial employing lymphocyte depletion, LD PT-Cy, and sirolimus is underway in patients with SCD who undergo haplo-HCT (Clinicaltrials.gov identifier NCT03077542).

### Supplementary information

Supplemental Figures with Figure Legends

Figure S1

Figure S2

Figure S3

Figure S4

## Data Availability

Data will be deposited in Figshare data repository 10.25444/nhlbi.25152866.
