# Supplementary material for: Low dose post-transplant cyclophosphamide and sirolimus induce mixed chimerism with CTLA4-Ig or lymphocyte depletion in an MHC-mismatched murine allotransplantation model
Source: Bone Marrow Transplant. 2024 Feb 12;59(5):615–24. doi: 10.1038/s41409-024-02237-y (PMC11073977; doi:10.1038/s41409-024-02237-y)
Supplement: Supplementary file 1 — Supplemental Figures with Figure Legends [file 41409_2024_2237_MOESM1_ESM.docx]

**Supplemental Figure Legends**

**
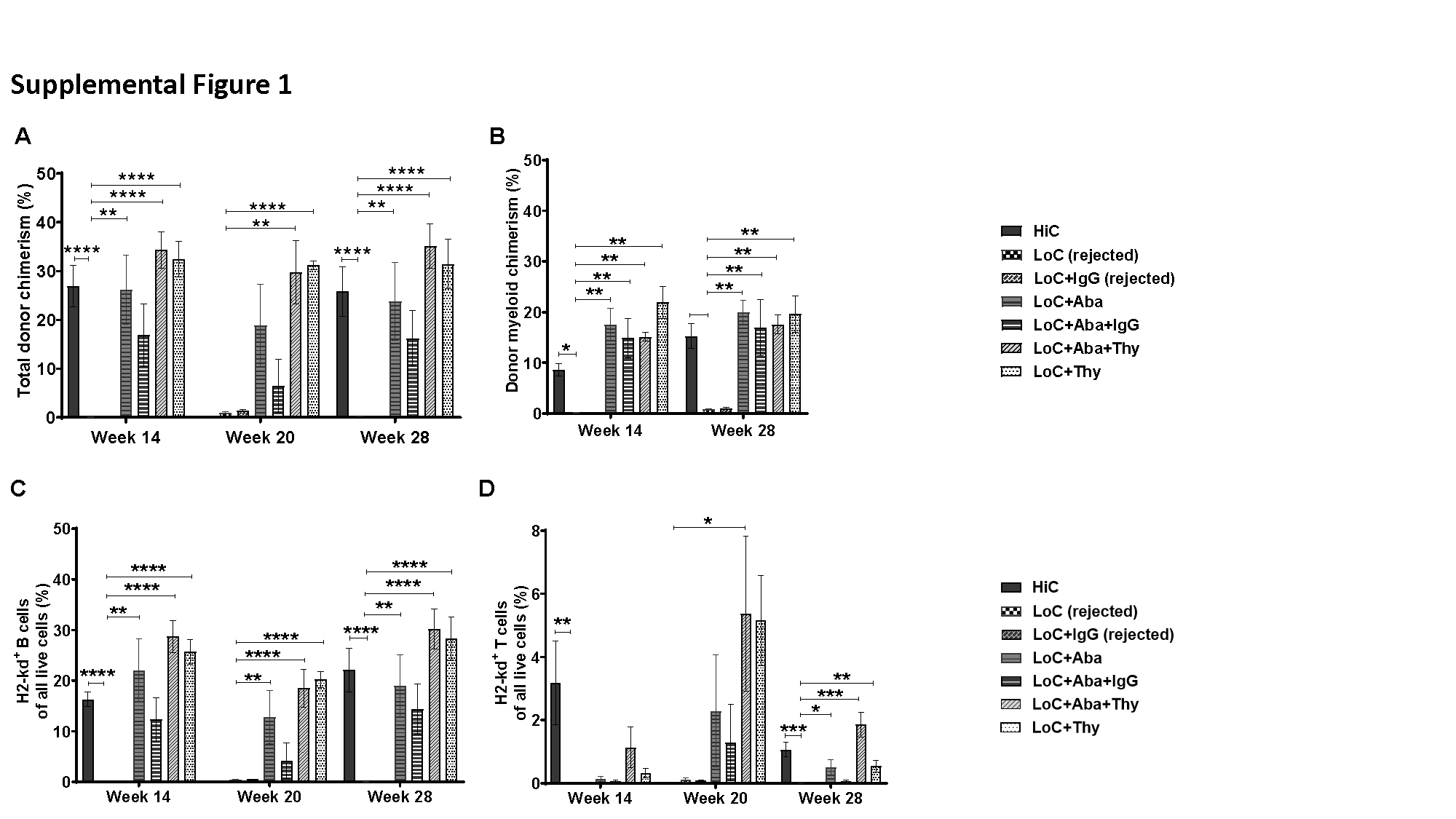
**

**Figure S1. The addition of anti-Thy1.2 with or without CTLA4-Ig led to prolonged donor chimerism levels of at least 20% from weeks 14- 28 PT in younger mice.**

Peripheral blood analysis at indicated time points of H2- kd^+^ total cells **(A)**, H2-K^d+^ CD11b^+^ myeloid cells **(B)**, H2- kd^+^ CD19^+^ B cells **(C)**, and H2- kd^+^ CD3^+^ T cells **(D)**. Data represent two experiments from n=3-9 mice (Mean $\pm$ SEM). (D* *P* < 0.05, ** *P* < 0.01, *** *P* < 0.001, and **** *P* < 0.0001 (unpaired two-tailed Student’s t-test).

**

**

**Figure S2. Representative flow plots depicting higher donor myeloid chimerism and total donor chimerism in younger and older mice within engrafted groups.**

Peripheral blood at 10 weeks PT of the younger mice (n=3-9, 6-8 weeks old), and week 8 PT of older male mice (n=5, 15-18 weeks old). H2- kd^+^ CD11b^+^ myeloid cells from younger recipient mice **(A)**, H2-K^d+^ total cells from younger recipient mice **(B)**. H2- kd^+^ total cells from older recipient mice **(C)**, H2-K^d+^ total cells from older recipient mice **(D)**. Representative flow plots from 3 independent experiments are shown.

**

Figure S3. No clear correlation between the T cell frequencies and the success or failure of engraftment in younger or older mice.**

Peripheral blood analysis at 4 weeks of host-derived H2- kb^+^ CD4^+^ T cells, H2- kb^+^ CD8^+^ T cells, and H2- kb^+^ CD4^+^ Tregs in younger mice **(A-C),**  and in older mice in **(D-F)**. Representative flow plot are represented for younger mice H2- kb^+^ CD4^+^ and H2- kb^+^ CD8^+^ T cells **(G**, left panel**)** and H2- kb^+^ CD4^+^ Tregs **(G**, right panel**)**. Older mice H2- kb^+^ CD4^+^ and H2- kb^+^ CD8^+^ T cells **(H**, left panel**)** and H2- kb^+^ CD4^+^ Tregs **(H**, right panel**).** Data represents from one-two experiments involving n=3-9 mice (younger) and n=5 mice (older) (Mean $\pm$ SEM)** *P* < 0.01, *** *P* < 0.001, and **** *P* < 0.0001 (unpaired t wo-tailed Student’s t-test).

**

**

**Figure S4. The addition of Thy1.2 facilitates splenic donor myeloid chimerism levels of greater than 30%.** Splenocyte analysis at 12 weeks PT of H2- kd^+^ total cells **(A)**, H2- kd^+^ CD11b^+^ myeloid cells **(B)**, H2-K^d+^ CD19^+^ B cells **(C)**, and H2- kd^+^ CD3^+^ T cells **(D)**. Data is obtained from representative experiment involving n=5 older mice/group (16-18 weeks old) (Mean $\pm$ SEM). ** *P* < 0.01, *** *P* < 0.001, and **** *P* < 0.0001 (unpaired two-tailed Student’s t-test).
