# Supplementary figures and images for: Low dose post-transplant cyclophosphamide and sirolimus induce mixed chimerism with CTLA4-Ig or lymphocyte depletion in an MHC-mismatched murine allotransplantation model
Source: Bone Marrow Transplant. 2024 Feb 12;59(5):615–24. doi: 10.1038/s41409-024-02237-y (PMC11073977; doi:10.1038/s41409-024-02237-y)

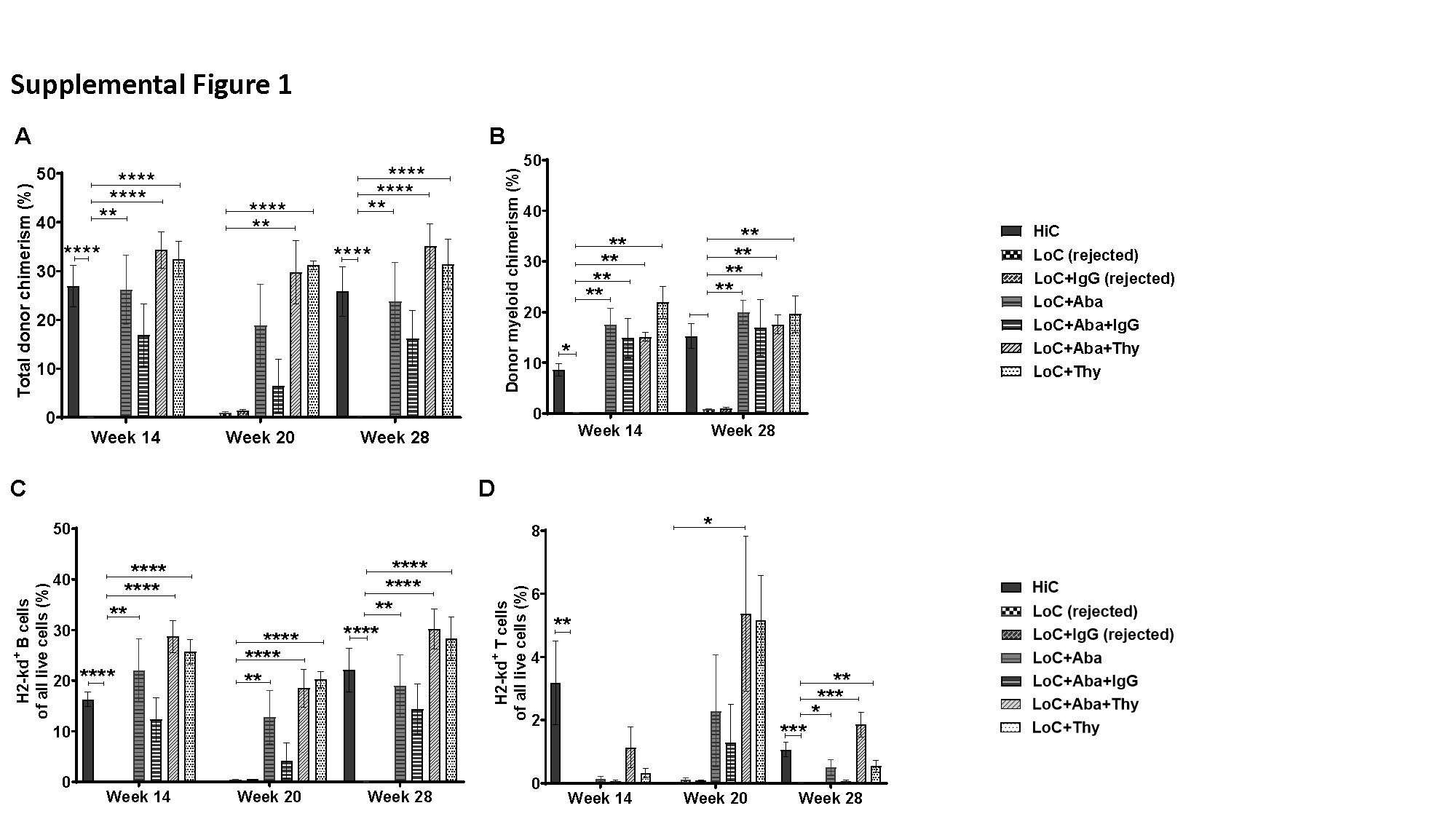

Supplement: Supplementary file 2 — Figure S1 [file 41409_2024_2237_MOESM2_ESM.tif]

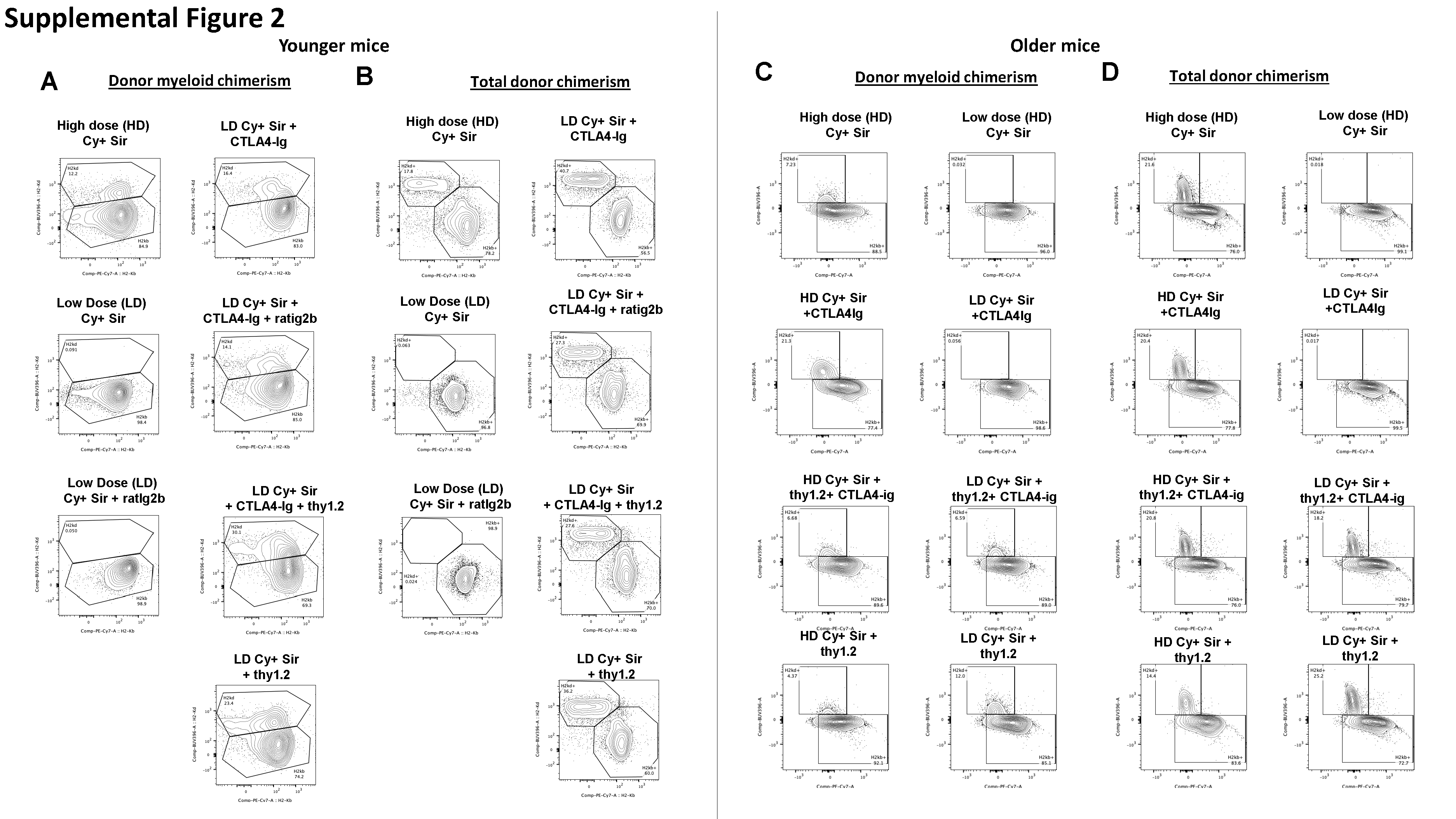

Supplement: Supplementary file 3 — Figure S2 [file 41409_2024_2237_MOESM3_ESM.tif]

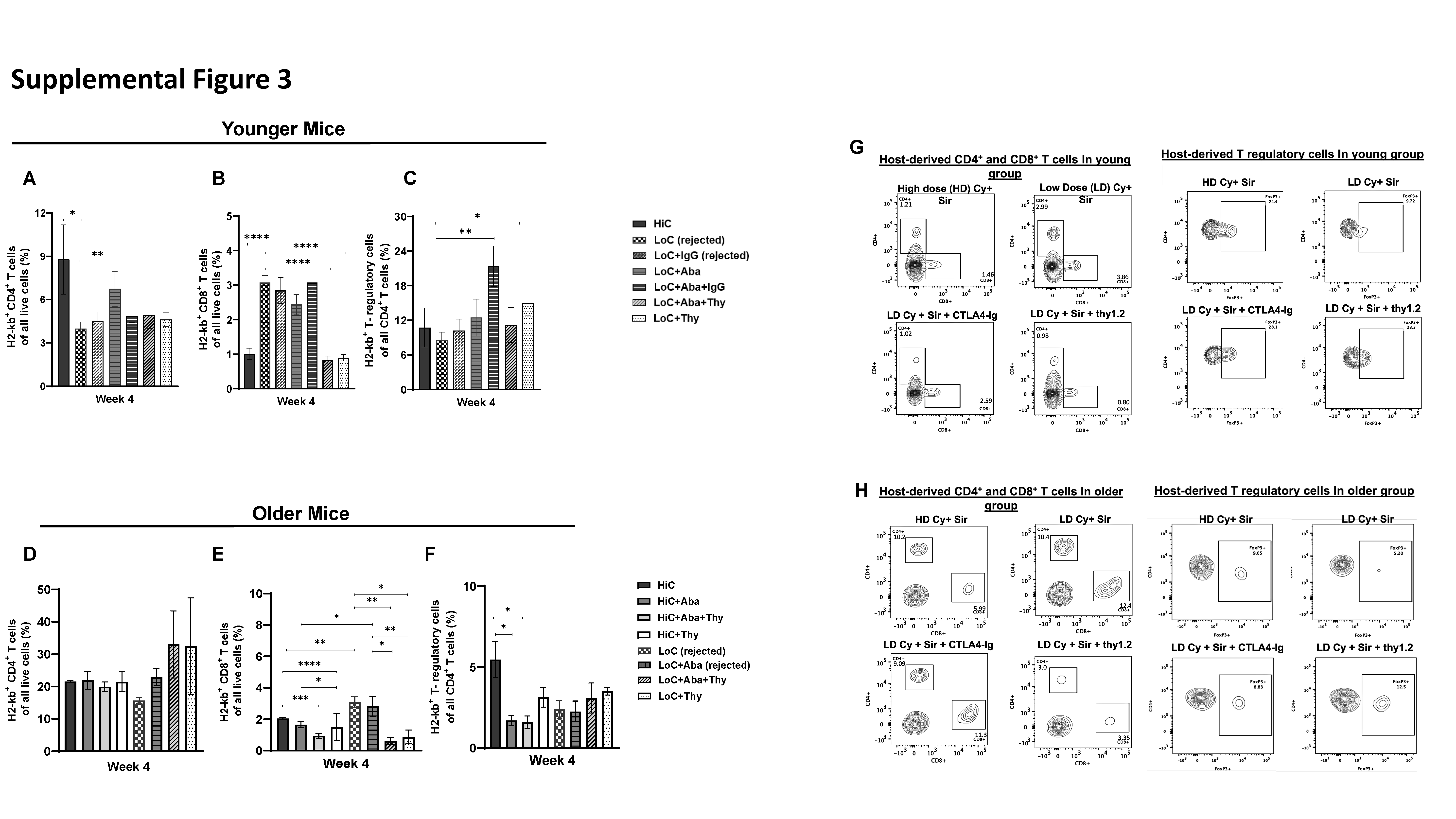

Supplement: Supplementary file 4 — Figure S3 [file 41409_2024_2237_MOESM4_ESM.tif]

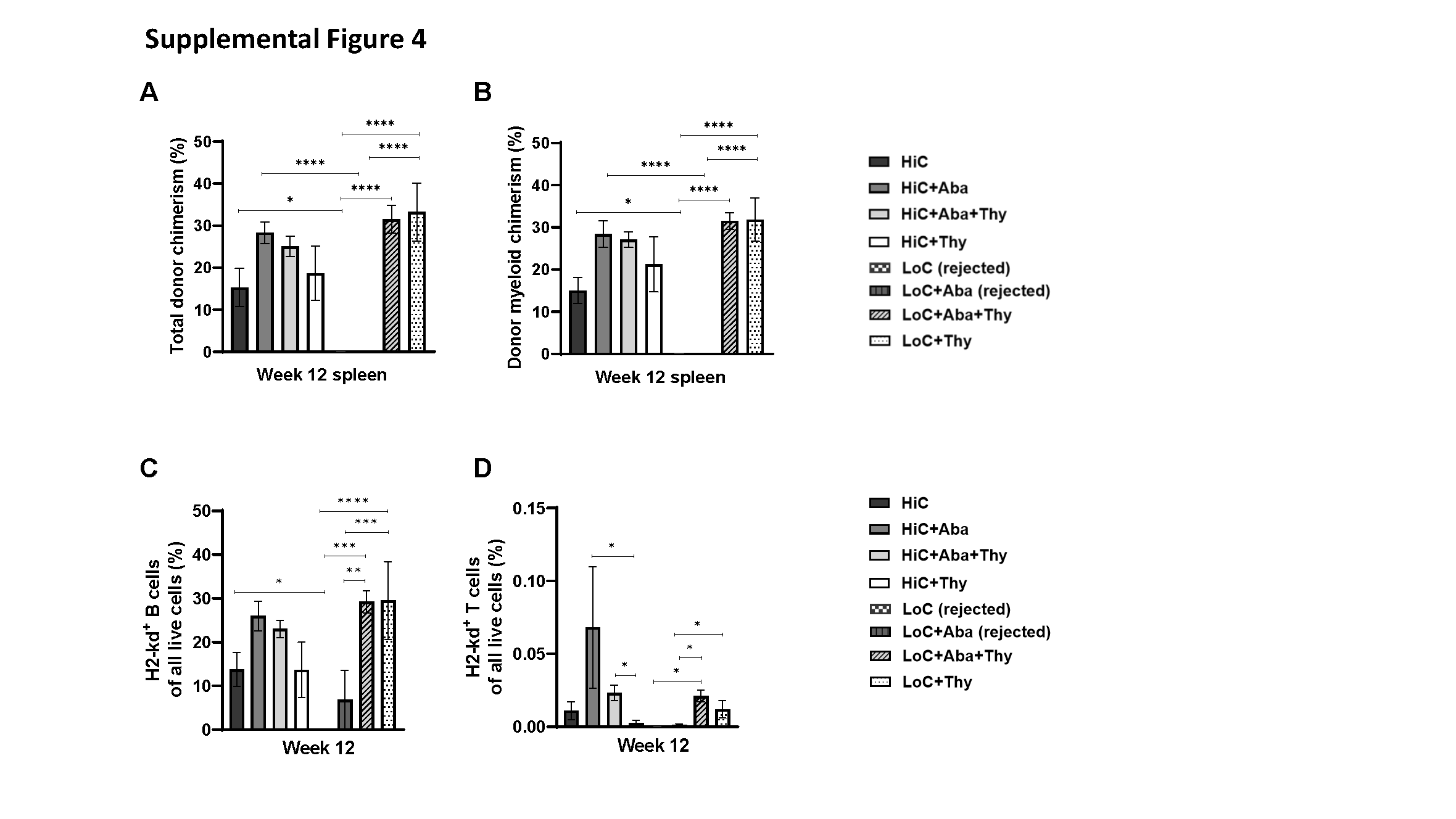

Supplement: Supplementary file 5 — Figure S4 [file 41409_2024_2237_MOESM5_ESM.tif]
